# Predicting Premature Video Skipping and Viewer Interest from EEG Recordings

**DOI:** 10.3390/e21101014

**Published:** 2019-10-19

**Authors:** Arno Libert, Marc M. Van Hulle

**Affiliations:** Department of Neurosciences, Laboratory for Neuro- & Psychophysiology, KU Leuven-University of Leuven, 3000 Leuven, Belgium

**Keywords:** emotion, engagement, valence, arousal, neuromarketing, button-press, multiscale version of sample entropy (MSE), interest, skipping

## Abstract

Brain–computer interfacing has enjoyed growing attention, not only due to the stunning demonstrations with severely disabled patients, but also the advent of economically viable solutions in areas such as neuromarketing, mental state monitoring, and future human–machine interaction. An interesting case, at least for neuromarketers, is to monitor the customer’s mental state in response to watching a commercial. In this paper, as a novelty, we propose a method to predict from electroencephalography (EEG) recordings whether individuals decide to skip watching a video trailer. Based on multiscale sample entropy and signal power, indices were computed that gauge the viewer’s engagement and emotional affect. We then trained a support vector machine (SVM), a k-nearest neighbor (kNN), and a random forest (RF) classifier to predict whether the viewer declares interest in watching the video and whether he/she decides to skip it prematurely. Our model achieved an average single-subject classification accuracy of 75.803% for skipping and 73.3% for viewer interest for the SVM, 82.223% for skipping and 78.333% for viewer interest for the kNN, and 80.003% for skipping and 75.555% for interest for the RF. We conclude that EEG can provide indications of viewer interest and skipping behavior and provide directions for future research.

## 1. Introduction

In recent years, brain–computer interfacing (BCI) has achieved a plethora of academic achievements, initially focusing on medical applications whilst progressively expanding towards other applications [1]. Popular BCI paradigms for controlling devices are the steady-state evoked potentials (SSEPs) [2,3] event-related potentials (ERPs) [4], and event-related desynchronization/synchronization (ERD/ERS) [5], mostly in combination with electroencephalography (EEG) [6]. EEG recordings have also been used in passive BCI applications for task engagement [7,8,9] and mental workload monitoring [10], and for emotion recognition [11]. The ability to monitor one’s mental state with EEG-based BCIs also raised interest from neuromarketers. Neuromarketing is concerned with the analysis and insight into human behavior in relation to markets and marketing exchanges, and hereto employs a variety of physiological and related recording techniques. The most advanced ones record brain activity in an attempt to chart customers’ mental responses to commercial messages and to monitor their true experience with a product. Specifically with regard to the former, it is important to find out which cognitive process(es) predict(s) a viewer’s decision to prematurely skip a commercial.

User interest is an ambiguous term which is likely to depend on several factors. In the open literature, interest is often linked to task engagement and gauged by the relative power of averaged full scalp EEG in beta, alpha, and theta bands [7,8,9]. Emotional responses have been gauged in terms of the relative power in beta and alpha bands of the F3 and F4 channels [12]. In [13], interest was defined as user experience and classified using EEG data of the entire video. Recent reports have shown the advantage of entropy-based methods of EEG analysis to discern emotional states, in particular the multivariate entropy ones as they are considered to better account for the temporal structure across channels [4,6,14,15,16].

When considering entropy for EEG analysis, one is factually aiming to quantify the degree of uncertainty or randomness present in the recordings. In previous works, entropy measures have been used in conjunction with multiscale signal transformations such as multivariate empirical mode decomposition (MEMD), which splits the EEG signal into frequency bands common to a set of scalp electrodes [16,17,18]. This has recently led to MEMD used in combination with multiscale entropy analysis, called MEMD enhanced MMSE [15,19]. The multiscale version of sample entropy (MSE) method relies on coarse graining, where the original signal is averaged over non-overlapping windows of increasing length and sample entropy applied on the ensuing temporal scale representations, providing a multiscale entropy analysis [20,21,22]. 

In our study, we extracted from EEG recordings indices of user engagement, valence, and arousal to predict viewer interest and the decision to prematurely skip the video. We used hereto entropy, as well as power-based features, and examined which ones contribute most to the prediction. Besides power, we computed sample entropy in the mu, beta, and theta frequency bands of the EEG signal in combination with coarse graining (multiscale sample entropy, [20,21]). The MEMD [18] method was also considered, but the presence of peaks in the frequency bands led to a suboptimal decomposition. A key difference with previous EEG studies on engagement is the use of entropy in separate frequency bands instead of their power. To the best of our knowledge there are no reports in the open literature on predicting video skipping behavior from EEG recordings.

## 2. Materials and Methods 

### 2.1. Experimental Setup

Four young adult participants were recruited (2 female, 2 left handed, aged 22–28 std 2.9), of which 1 never participated in an EEG study. Participants read and, when they agreed, signed the consent form approved by the ethical committee of our university hospital UZLeuven (S52112, approved on 29 January 2010). All participants had normal or corrected-to-normal vision.

EEG data was recorded utilizing 32 active Ag/AgCl electrodes, evenly distributed over the scalp according to the international 10–20 system, at a sampling rate of 2000 Hz using a SynAmps RT EEG device. Ground and reference electrodes were placed at AFz and FCz, respectively. Electrooculography (EOG) data were recorded using electrodes placed above and below the left eye for post-hoc artifact removal.

The experiment had each participant watch a video with length of approximately 1 min. The videos were displayed on a VIEWPixx monitor with a 120 Hz refresh rate, with participants seated approximately 70 cm from the screen at a 30 Hz framerate. The left hand was positioned on the space bar to perform the skipping decision, whilst the right hand was rested upon the numpad for question responding to minimize motor activity during recordings. Participants were informed to type 1 for yes and 0 for no. Participants were instructed to press the space bar of the computer keyboard to stop the video. Immediately after the button press, or at the end of the video when it was watched in its entirety, the participant was prompted with 2 yes/no questions: “Was the video interesting?” and “Have you watched this video before?”. Prior to each video, participants were instructed to refrain from blinking as much as possible, to avoid EEG eye artefacts, and to focus on the white cross on the screen for 3 s, during which baseline EEG activity was recorded (see further). In total, each participant watched 45 thematically varied videos, interrupted by a brief break every 15 videos to avoid fatigue. Note that the 45 videos were not the same for each participant. The list of used videos is provided in the Supplementary Materials.

### 2.2. Data Preprocessing

**Figure 1:**
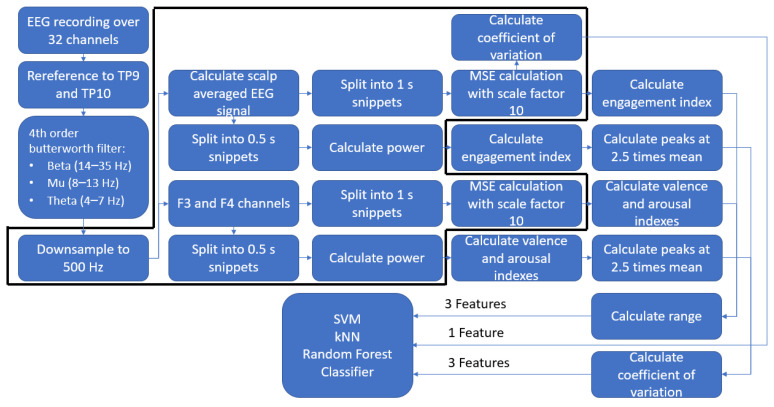
Overview of the processing pipeline. Note that each step within the black outline applies to each frequency band. Abbreviations: MSE, multiscale version of sample entropy; SVM, support vector machine; kNN, k-nearest neighbor; EEG, electroencephalography.

All data preprocessing was performed offline in Matlab 2018b [23]. An overview of the processing pipeline can be found in Figure 1. The raw EEG signal was re-referenced to the average of the mastoid signals (TP9 and TP10). Then, from the EEG recordings, the 3 s baseline activity was extracted, its average amplitude computed and subtracted from the subsequent video-related EEG recordings from onset until button press, or until the end of the video when watched entirely. After that, the baseline-corrected EEG recordings were cut into 1 s epochs for the entropy calculation and 500 ms epochs for power calculation. Epochs were filtered by a channel-wise bandpass filter in the theta (4–7 Hz), mu (8–13 Hz), and beta (14–35) bands and downsampled to 500 Hz. When calculating the engagement index (see further), for both the power and entropy cases, the EEG signals of all.

Channels were averaged into a scalp-averaged EEG signal. However, when calculating the arousal and valence indices, only the EEG signals of the F3 and F4 channels were used and kept separate. 

### 2.3. Analysis

#### 2.3.1. MSE

A signal’s entropy is a measure for the amount of information it carries. A popular metric is sample entropy [24]:

(1) $$SampEn\left(r,m,N\right)=-log\frac{A^{m+1}\left(r\right)}{B^{m}\left(r\right)},$$

with *r* the tolerance level, *B^m^* the probability that two sequences with length *m* are similar within *r*, and *A^m^*^+1^ the probability that these two sequences remain similar within *r* when extending their length to *m*+1. The tolerance level was set to 0.15*std of the 1 s partition’s amplitude.

For the multiscale version of sample entropy (MSE), we relied on coarse graining [22], where sample entropy was calculated for the original signal, as well as for each of the scaled versions. The time series used as input for the MSE method is as follows:

(2) $$y_{j}^{\left(\tau \right)}=\frac{1}{\tau }\sum_{i=\left(j-1\right)\tau +1}^{j\tau }x_{i}\;with\;1\leq j\leq \frac{N}{\tau },$$

where $y_{j}^{\left(\tau \right)}$ is the sample entropy calculated for step *j* < τ, with *τ* = 10 the scale factor; for *τ* = 1, the time sequences comprise 1000 ms or 500 samples, and for *τ* = 10, 100 ms or 50 samples. Finally, the entropies of these 10 scales were averaged, yielding a univariate entropy measure. Note that the univariate entropy measure was calculated on 1 s partitions of the scalp-averaged EEG, as well as separately for the F3 and F4 EEG signals also on 1 s partitions.

#### 2.3.2. Engagement, Arousal, and Valence Indices

Engagement was calculated using the formula [7]:

(3) $$Engagement=\;\frac{Beta}{Mu+Theta},$$

with Beta, Mu, and Theta represent the power in the corresponding frequency bands of the scalp-averaged EEG. Arousal and valence were calculated using the formula [7]:

(4) $$Arousal=\;\frac{Beta\;F3+Beta\;F4}{Alpha\;F3+Alpha\;F4}\;\mathrm{and}$$

(5) $$Valence=\frac{Alpha\;F4}{Beta\;F4}-\;\frac{Alpha\;F3}{Beta\;F3},$$

with Beta and Alpha the power in the respective frequency bands, computed for the F3 and F4 channels’ EEG signals. In addition, the engagement index was also calculated using the entropy in the respective frequency bands of the scalp-averaged EEG, and likewise the valence and arousal indices, but then for the F3 and F4 channels.

#### 2.3.3. Features

Features were developed for the classifier to capture the viewer’s engagement, arousal, and valence from the recorded EEG signal. We opted for 9 features, 6 based on entropy and 3 on power. 

The first three entropy-based features are the range (maximum–minimum) spanned by the coefficient of variation (CV) during the video of the beta, mu, and theta frequency bands. The CV was calculated as follows, on 1 s epochs:

(6) $$CV=\;\frac{standard\;deviation}{mean}.$$

These features were further abbreviated as RCVB (range of CV beta band), RCVM (range of CV mu band), and RCVT (range of CV theta band).

The three other entropy-based features were taken as the range (maximum–minimum) of the entropy-based engagement, arousal, and valence indices. These were further abbreviated as REEI (range entropy engagement index), REAI (range entropy arousal index), and REVI (range entropy valence index). 

The three power-based features were based on the contrasted coefficient of variation (CV2) of the absolute difference in successive peaks calculated for the power-based engagement, arousal, and valence indices:

(7) $$CV2=\;\frac{1}{n-1}\sum_{i=1}^{n-1}\frac{2*\left|peak_{i+1}-peak_{i}\right|}{peak_{i+1}+peak_{i}},$$

where a peak has to be larger than 2.5 times the mean of the corresponding index. These features were abbreviated as CV2EI (CV2 engagement index), CV2AI (CV2 arousal index), and CV2VI (CV2 valance index).

#### 2.3.4. Classification 

To test the validity of the selected features for the classification task, we utilized a gradient ascent support vector machine (SVM) [25], a k-nearest neighbor (kNN) with the cityblock heuristic (k = 10), and a random forest (RF) classifier, all of which are available in Matlab. The classifiers were trained to find distinguishing patterns for the skipped/not skipped cases and the interested/not interested cases. In order to validate the classifiers, we opted for a leave-one-subject-out cross-validation. In addition, to investigate the functionality of the individual features, we performed a pseudorandom holdout cross-validation of 10% of the total data (all subjects and watched videos), repeated the process 500 times, and reported the averaged classification results.

It is important to note that, when the viewer was interested in the video, the output of the corresponding classifier was set to 1, whilst not interested, it was set to −1 (binary classification problem). Similarly, when a video was not skipped, the output of the corresponding classifier was set to 1, whilst when skipped it was set to −1.

## 3. Results

### 3.1. Skipping and Interest Prediction Accuracies

Leave-one-subject-out cross-validation (LoSOCV) results are listed in Table 1 for the SVM, kNN, and RF classifiers. The columns indicate the used classifier and the rows the subjects whose data was used for testing the classifiers prior trained on data from the remaining subjects. The kNN classifier performs, on average, the best for both the skipped and interest predictions. The results show a larger accuracy for skipping than for interest for all classifiers. Several hypotheses can be developed to explain these results. The movement-related ERD is expected to occur in the last few seconds before button-pressing, thus the skipped videos are expected to exhibit a decrease in power in the mu and beta bands over the motor regions of the scalp (central EEG channels) [5]. Another explanation for the difference is that interest is more ambiguous than pressing a button and the proposed features could only partially capture what defines interest for a given viewer. To get a better understanding of what affects the prediction, we took a closer look at the contribution of the proposed features, as discussed next.

### 3.2. Feature Relevance

**Figure 2:**
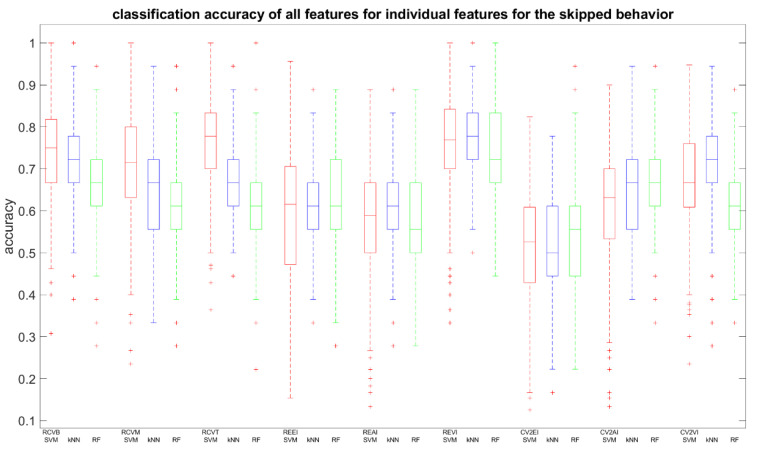
Boxplots summarizing accuracies of skipping prediction using individual features for the SVM (red), kNN (blue), and RF (green) classifiers. Note: Outliers correspond to the distribution in its column.

**Figure 3:**
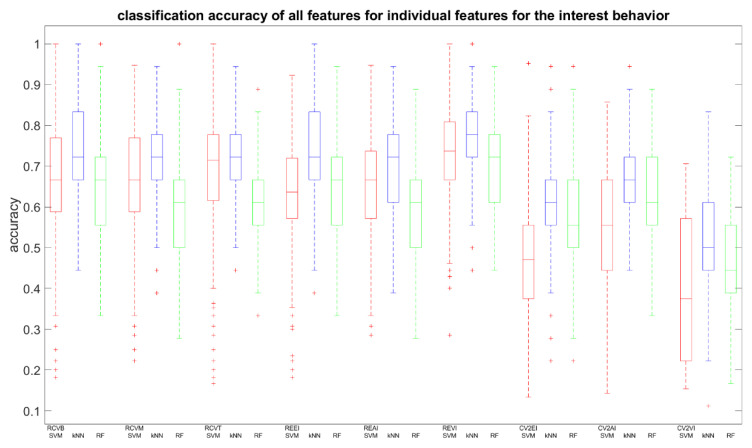
As in Figure 3, but for interest prediction.

We retrained the SVM, kNN, and RF classifiers, but now based on each feature individually, to assess the impact on the two prediction accuracies (feature relevance). Averaged prediction accuracies and standard deviations for skipping and interest are summarized in Table 2a,b and the distribution expressed in boxplots in Figure 2 and Figure 3, respectively. From Table 2a, we observe that the highest scores are for the RCV features and the REVI feature, which reached >70% accuracy. Compared to Table 2b, we see that only RCVT and REVI reached similar results. A Bonferroni corrected (for multiple comparisons, α = 0.05/9) pairwise Wilcoxon signed rank test was used to analyze whether one feature performed equally well for both cases, with H0 being the compared populations share a distribution with an identical median and therefore perform equally well. In Table 2, we can deduce that different classifiers benefit from different features. The SVM for the skipping case performed best for the RCVB, RCVM, RCVT, and REVI features (accuracy: >70%). For the interest case, only REVI outperformed, although no feature distribution shared median accuracies (*p* < 0.00556). When inspecting results for the kNN, we can report an interesting observation. Although the accuracy for all features is lower for the interest case compared to the skipping case, we observe that the performance for individual features has an opposite behavior. Only REVI (*p* = 0.1603) showed an equal kNN performance between cases. The RF classifier showed equal performance for the RCVB (*p* = 0.02251), RCVT (*p* = 0.82852), and REAI (*p* = 0.20695). An important observation is the low performance of CV2EI for the SVM and CV2VI for the SVM and RF for interest prediction, lower than 50%, meaning that it failed to discern the two classes and, therefore, does not qualify as a distinctive feature. However, the same does not pertain to skipping prediction. 

**Figure 4:**
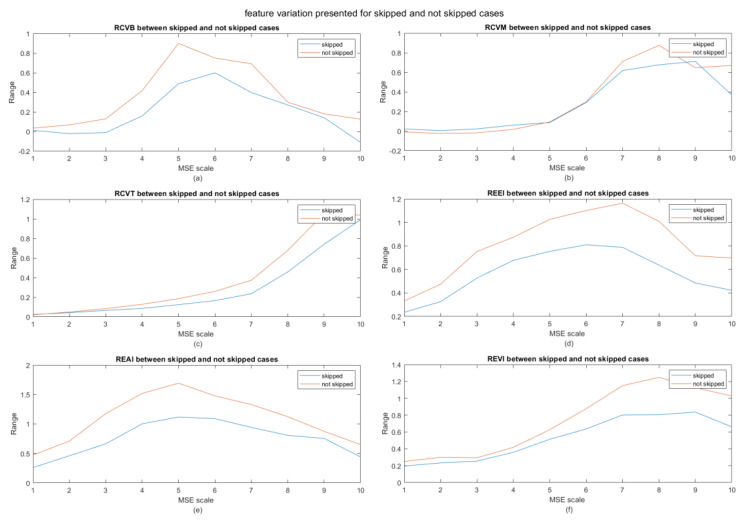
Difference between features in skipped and non-skipped cases. (**a**) RCVB, (**b**) RCVM, (**c**) RCVT, (**d**) REEI, (**e**) REAI, and (**f**) REVI.

**Figure 5:**
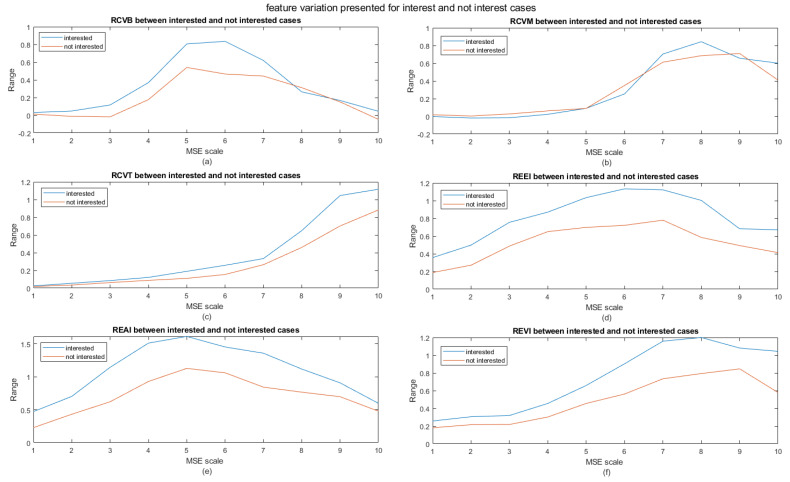
Idem to Figure 5 but for interest and not interest cases. (**a**) RCVB, (**b**) RCVM, (**c**) RCVT, (**d**) REEI, (**e**) REAI, and (**f**) REVI.

We also looked at the contribution of the power and entropy features (except for the CV2 features which did not perform well) as a function of coarse graining scale (MSE scale). The results for the skipped/not skipped cases are shown in Figure 4 and for the interest/not interest cases in Figure 5. RCVB and RCVT are more discerning compared to RCVM. Another interesting observation is that the range appears to peak at different MSE scales. When observing the differences in features between the interested and not interested cases, we observe a general increase in range for the interested cases compared to the not interested ones. The plots for the skipped case show an increased range for the not skipped cases compared to the skipped cases. From this, we can logically conclude that, when a user is not interested in a video, he/she will most likely skip the video; however, this is not always the case (see Supplementary Materials). It seems that, when a user is not skipping a video or is interested in a watching it, the range increases, which in turn is indicative of larger fluctuations in overall information content.

## 4. Discussion

We have shown that a classifier can be trained on EEG recordings to predict a viewer’s decision to prematurely skip watching a video and his/her expression of interest in the video. We considered hereto the previously established engagement, arousal, and valence indices based on power in beta, mu, and theta bands [7], which we extended to entropy. As features, we took the range spanned by the coefficient of variation (CV) in beta, mu, and theta band power, the range spanned by entropy-based engagement, arousal, and valence indices, and the power-based contrasted CV (CV2) in these indices. We showed that the outcome of these features could be linked to the viewer’s interest when engaged in video viewing. To our knowledge, no previous reports exist on video skipping prediction from EEG. 

Although the classification algorithms proved accurate in predicting a viewer’s skipping and interest behavior, the physiological meaning behind the underlying responses still eludes us and calls for a more in-depth study, possibly supplemented by results from source localization. In previous works [7,8,9], the mu and theta bands were directly linked to disinterest or disengagement, whilst the beta band to interest or engagement. This is supported by our results, as they showed an increased range spanned by the entropy-based features for the interesting videos compared to the skipped videos. The increased range can be explained by increased information content of the beta band and/or decreased information content of the mu and theta bands. 

We are aware of several shortcomings in our work. First, in hindsight, the experiment could have been better controlled. We could have considered a longer eyes-open/eyes-closed baseline condition (up to 5 min) as it could turn out to be better suited for baseline-correcting our recordings. Also, the user was not given the option to choose a video he/she could be interested in. Second, connected to the former, some of the videos could be topic-wise similar to what the viewer has seen before and therefore trigger a sense of déjà vu (e.g., BBC newscasts). Third, several videos were expertly designed to trigger an emotional response (e.g., trailer from the movie “IT”, horror genre) whilst others not (e.g., landscapes videos, relaxing). Fourth, responses could vary heavily between viewers. Since the set of videos watched by the subjects were not exactly the same, it is difficult to discover responses shared by them. Fifth, the population was too small to warrant firm conclusions. However, the reported study was a pilot to investigate feasibility.

The relevance for the neuromarketer is clear: It would be valuable to determine which scene in a commercial eventually leads to premature skipping, even more so when the used technique would be based on brain responses instead of verbal post factum accounts—because the former is unbidden and immediate, while the latter perhaps socially corrected and prone to the recency effect.

## Figures and Tables

**Table 1:** Accuracies for skipping and interest for the SVM, kNN, and random forest (RF) classifiers when using all features. Each column corresponds to the respective classifier, whilst each row corresponds to which subject was left out.

Skipped	SVM	kNN	RF	Interest	SVM	kNN	RF
s1	76.60	80.00	86.67	s1	85.00	82.22	84.44
s2	96.97	84.44	77.78	s2	64.52	80.00	75.56
s3	65.12	88.89	77.78	s3	61.54	97.78	80.00
s4	64.52	75.56	77.78	s4	82.14	53.33	62.22
Average	75.8025	82.2225	80.0025	Average	73.3	78.3325	75.555

**Table 2:** Average accuracies and standard deviations for skipping (**a**) and for interest (**b**) when using individual features.

(**a**)						
**Skipped**	**SVM**	**sd**	**kNN**	**sd**	**RF**	**sd**
RCVB	0.734	0.113	0.712	0.099	0.654	0.105
RCVM	0.705	0.118	0.647	0.098	0.621	0.105
RCVT	0.759	0.109	0.684	0.099	0.615	0.105
REEI	0.585	0.156	0.618	0.099	0.618	0.106
REAI	0.578	0.144	0.608	0.108	0.583	0.106
REVI	0.756	0.117	0.775	0.088	0.735	0.103
CV2EI	0.518	0.131	0.516	0.107	0.544	0.115
CV2AI	0.611	0.133	0.643	0.099	0.665	0.100
CV2VI	0.676	0.113	0.723	0.099	0.602	0.104
(**b**)						
**Interest**	**SVM**	**sd**	**kNN**	**sd**	**RF**	**sd**
RCVB	0.676	0.131	0.740	0.096	0.640	0.105
RCVM	0.670	0.137	0.719	0.102	0.591	0.113
RCVT	0.693	0.139	0.728	0.095	0.614	0.107
REEI	0.641	0.116	0.736	0.106	0.645	0.106
REAI	0.654	0.122	0.696	0.099	0.592	0.108
REVI	0.725	0.121	0.766	0.091	0.694	0.098
CV2EI	0.465	0.138	0.607	0.107	0.577	0.107
CV2AI	0.548	0.150	0.683	0.098	0.629	0.106
CV2VI	0.396	0.177	0.526	0.106	0.463	0.108

## Supplementary Materials

The following are available online at https://www.mdpi.com/1099-4300/21/10/1014/s1.
